# Assessment of central venous catheter colonization using surveillance culture of withdrawn connectors and insertion site skin

**DOI:** 10.1186/s13054-016-1201-0

**Published:** 2016-02-02

**Authors:** María Jesús Pérez-Granda, María Guembe, Raquel Cruces, José María Barrio, Emilio Bouza

**Affiliations:** 1 CIBER Enfermedades Respiratorias-CIBERES (CB06/06/0058), Madrid, Spain; 2 Cardiac Surgery Postoperative Care Unit, Hospital General Universitario Gregorio Marañón, Madrid, Spain; 3 Instituto de Investigación Sanitaria Gregorio Marañón (IISGM), Dr. Esquerdo, 46, 28007 Madrid, Spain; 4 Department of Clinical Microbiology and Infectious Diseases, Hospital General Universitario Gregorio Marañón, Dr. Esquerdo, 46, 28007 Madrid, Spain; 5 Medicine Department, School of Medicine, Universidad Complutense de Madrid, Pza. Ramón y Cajal, s/n Ciudad Universitaria, 28040 Madrid, Spain

**Keywords:** Central venous catheters, Surveillance, Skin cultures, Closed needleless connectors, Colonization, Catheter-related bloodstream infection

## Abstract

**Background:**

Culture of catheter hubs and skin surrounding the catheter entry site has a negative predictive value for catheter tip colonization. However, manipulation of the hub for culture requires the hubs to be swabbed, introducing potential dislodging of biofilm and subsequent migration of microorganisms. Hubs are usually closed with needleless connectors (NCs), which are replaced regularly. Our objective was to evaluate whether culture of flushed withdrawn NCs is an alternative to hub culture when investigating central venous catheter colonization.

**Methods:**

The study population comprised 49 intensive care unit patients whose central venous catheters had been in place for at least 7 days. Cultures of NCs and skin were obtained weekly.

**Results:**

We included 82 catheters with more than 7 days’ indwelling time. The catheter tip colonization rate was 18.3 % (15/82). Analysis of skin and NC cultures revealed a 92.5 % negative predictive value for catheter colonization. Three episodes of catheter-related bloodstream infection (C-RBSI) occurred in patients with colonized catheters.

**Conclusion:**

Surveillance of NC and skin cultures could help to identify patients at risk for C-RBSI.

## Background

Catheter-related bloodstream infection (C-RBSI) is a severe condition with high rates of associated morbidity and mortality [1, 2]. It occurs after catheter tip colonization by microorganisms progressing along both the inner and outer surface of the catheter [3, 4]. Diagnosis of catheter tip colonization is confirmed by culturing the catheter tip after withdrawal but may be anticipated by conservative methods based on superficial cultures of hubs and the skin surrounding the catheter entry site [5]. Catheter colonization is considered a harbinger of C-RBSI and may be used to identify an at-risk population [3, 4, 6–8]. However, hub culture requires the hubs to be swabbed, introducing potential dislodging of biofilm and subsequent migration of microorganisms [9, 10].

At present, most catheter hubs are occluded by closed needleless connectors (NCs), which, according to the manufacturer’s instructions, must be replaced regularly [11]. The aim of our study was to evaluate whether culture of flushed withdrawn connectors is an alternative to hub culture when investigating catheter colonization.

## Methods

### Setting

The major heart surgery intensive care unit (MHS-ICU) in our hospital is a 14-bed post-surgical unit for all adult patients who have undergone a major cardiac surgical procedure. The study population comprised patients who were admitted to the MHS-ICU during the study period (12 Jan. 2015 to 31 May 2015) and whose central venous catheter (CVC) had been in place for at least 7 days after insertion. All catheter tips were sent for culture irrespectively of the reason for withdrawal. We excluded patients whose catheter tip had inadvertently not been sent for culture.

### Laboratory procedures

**Fig. 1:**
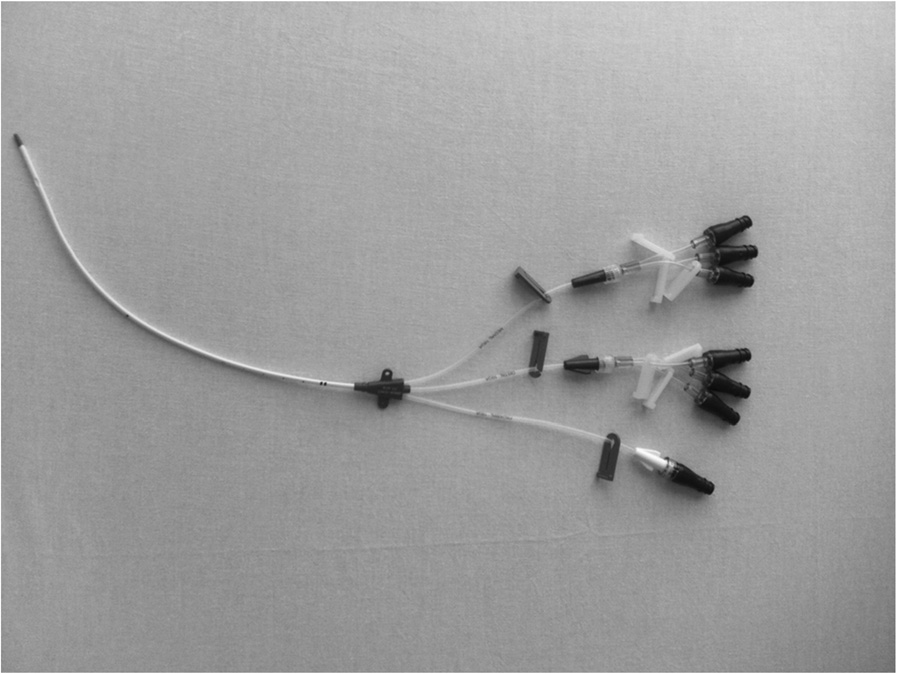
Central venous catheter

**Fig. 2:**
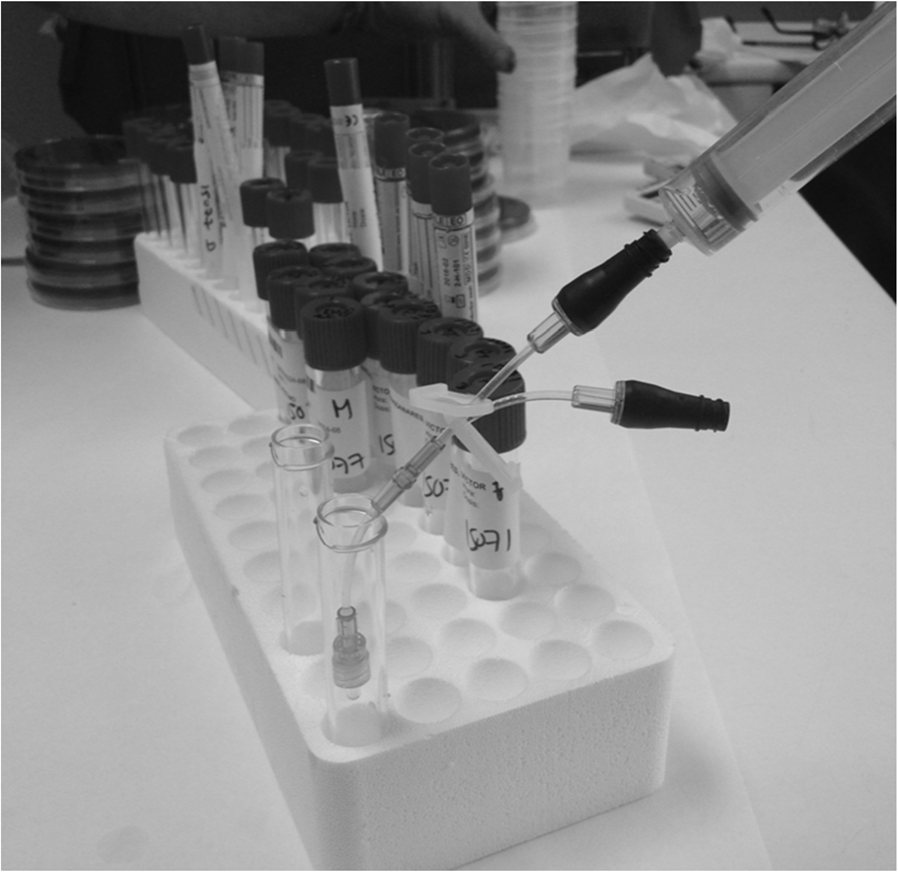
Laboratory procedure for needleless connector (NC) flushing

In accordance with the instructions of the manufacturer, NCs (CLAVE systems; ICU Medical, Inc., San Clemente, CA, USA) were changed every 7 days and cultured. Skin cultures were taken simultaneously from the catheter entry site (a swab rubbed into the surface of 1–2 cm around the catheter insertion site) when the NC was withdrawn and processed following standard semi-quantitative microbiological techniques [5]. All NCs from a single catheter lumen were individually flushed with 100 μl of brain-heart infusion and this flush was cultured into a blood agar plate (Figs. 1 and 2). We considered the lumen colonized when at least one culture was positive. The number of NCs cultured varied depending on the number of lumens per catheter (1–5 lumens).

Catheter tips were withdrawn when clinically indicated and cultured immediately by using the roll-plate (Maki) technique or sonication onto a blood agar plate or both [12]. The microorganisms recovered were identified by using standard microbiological methods and matrix-assisted laser desorption/ionization-time of flight (MALDI-TOF) [13]. We used a pre-established protocol to record patient characteristics, underlying diseases, comorbidities, severity of illness scores, and blood culture results at the time of catheter withdrawal.

### Definitions

#### Catheter tip colonization

We isolated either at least 15 colony-forming units (CFU) per segment using the semi-quantitative Maki technique or at least 100 CFU per segment using the sonication method [5].

#### Skin colonization

We isolated at least 15 CFU per plate in the semi-quantitative culture [5].

#### Closed needleless connector colonization

We isolated at least 10 CFU per connector in at least one connector in the qualitative culture.

#### Lumen colonization

Lumen colonization was considered to have occurred when culture of at least one NC from one lumen was positive at any time during surveillance.

#### C-RBSI

We considered a C-RBSI episode to be confirmed when the same microorganism was isolated both in peripheral blood cultures (obtained 7 days before or after catheter withdrawal) and from the catheter tip [5]. The gold standard for catheter colonization was positivity of the catheter tip culture by using either the semi-quantitative Maki technique or the quantitative sonication method [5]. To calculate the validity values of skin and NC cultures for predicting catheter colonization, we used a positive catheter tip with at least 15 CFU per plate of any microorganism as the gold standard.

### Statistical analysis

Continuous variables are expressed as the mean (standard deviation, or SD) or median (interquartile range), and categorical variables as percentages with a 95 % confidence interval (CI). Categorical variables were evaluated by using the chi-squared or two-tailed Fisher exact test. Statistical significance was set at a *P* value of less than 0.05 (two-tailed).

We calculated the validity values of the closed NC culture by comparing it with the gold standard of colonization. The sensitivity, specificity, and positive and negative predictive values with their 95 % CIs were calculated by using EPIDAT 3.1. Accuracy was defined as the sum of true-positive and true-negative results.

Kaplan-Meier survival curves and the log-rank test were used to compare the time to positivity of the colonization of the first positive skin or NC culture (or both) between colonized and non-colonized catheters. The statistical analysis was performed by using IBM SPSS Statistics for Windows version 21.0 (IBM Corporation, Armonk, NY, USA).

### Ethics

The study was approved by the local ethics committee, and the ethics committee waived the need for informed consent.

## Results

We included 82 catheters with at least 7 days’ indwelling time from 49 patients. Mean (SD) age was 64.7 (12.6) years. The main underlying conditions were congestive heart failure (57.1 %), diabetes mellitus (40.8 %), and other diseases (2.1 %). The overall mean (SD) comorbidity index, Acute Physiology and Chronic Health Evaluation II (APACHE II) score at inclusion, and EuroSCORE were, respectively, 3.4 (4.3), 8.4 (3.0), and 6.9 (2.4). The main reason for catheter withdrawal was end of use (65.9 %), followed by suspicion of infection (24.4 %), and a miscellany of other reasons (9.8 %). We confirmed three episodes of C-RBSI (2.5 episodes per 1000 catheter days). Additional patient and catheter data are shown in Table 1. The crude mortality rate of the selected population under study was 24.4 %. We did not find statistically significant differences between the use of parenteral nutrition and catheter colonization (*P* = 0.34).

**Table 1:** Main characteristics of patients and catheters

Characteristic	N (%)
Patients (n = 49)	
Mean (SD) age, years	64.7 (12.6)
Sex male/female	30/19
Underlying conditions	
Myocardial infarction	4 (8.2)
Congestive heart failure	28 (57.1)
Central nervous system (ACVA)	10 (20.4)
Chronic obstructive pulmonary disease	9 (18.4)
Diabetes mellitus	20 (40.8)
Peptic ulcer disease	7 (14.3)
Peripheral vascular disease	3 (6.1)
Renal dysfunction	9 (18.4)
Mean (SD) EuroSCORE^a^	6.9 (2.4)
Mean (SD) comorbidity index (Charlson criteria)	3.4 (4.3)
Non-fatal underlying disease (McCabe criteria)	39 (79.6)
Mean (SD) APACHE II at inclusion	8.4 (3.0)
Median (IQR) length of ICU stay, days	13.0 (8.0–28.0)
Crude mortality	11 (24.4)
Catheters (n = 82)	
Type of catheter	
Non-tunneled central venous catheter	62 (75.6)
Guidewire	20 (24.4)
Location	
Jugular	78 (95.1)
Subclavian	4 (4.9)
Total parenteral nutrition	21 (25.6)
Reasons for catheter withdrawal	
End of use	54 (65.9)
Suspicion of infection	20 (24.4)
Other	8 (9.8)
Median (IQR) indwelling time, days	11.0 (8.0–20.0)
Total number of catheter days	1215
Catheter colonization	15 (18.3)
Catheter colonization, density per 1000 catheter-days	12.3
C-RBSI episodes	3 (3.7)
C-RBSI per 1000 catheter days	2.5

*SD* standard deviation, *IQR* interquartile range, *ICU* intensive care unit, *C-RBSI* catheter-related bloodstream infection, *ACVA* acute cerebrovascular accident. ^a^EuroSCORE: European System for Cardiac Operative Risk Evaluation

**Fig. 3:**
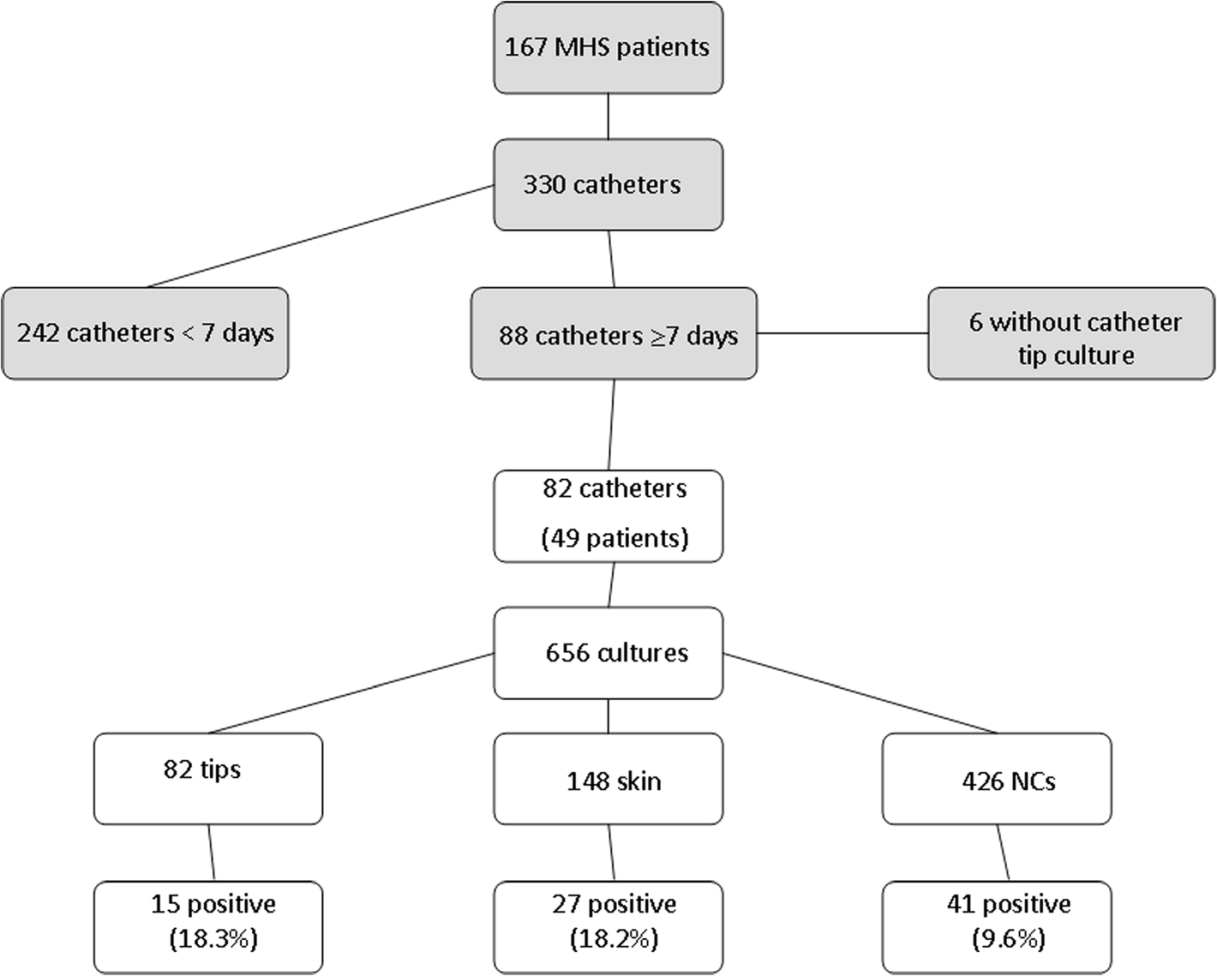
Flowchart showing how samples were included

We collected a total of 656 cultures (82 catheter tips, 148 skin cultures, and 426 NCs) (Fig. 3). The 82 catheters were evaluated between days 8 and 20 after insertion (median of 11 days). A median of 3 (3–6) NC cultures were performed for each catheter.

The catheter tip colonization rate was 18.3 % (15/82). The culture results for the skin and lumens are summarized in Fig. 3. Positive results were detected in 18.2 % (27/148) of the skin cultures, and 9.6 % (41/426) of the lumen cultures were positive. Positive skin or lumen culture results or both were not detected in 40 (48.8 %) of the 82 catheters. In the remaining 42 (51.2 %), the skin or lumens or both were positive at least once.

### Prediction of catheter colonization and C-RBSI by skin or NC culture or both

Analysis of catheter colonization and C-RBSI by considering skin and NC culture colonization together as a single test showed 80.0 % sensitivity and 92.5 % negative predictive value. In addition, a negative result for all lumen cultures (all NC cultures) had a negative predictive value of 100 % for C-RBSI (Table 2).

**Table 2:** Validity values of skin and needleless connector cultures for prediction of catheter colonization and catheter-related bloodstream infection

Cultures	S %	SP %	PPV %	NPV %	Validity index	Prevalence	LR^+^	LR^−^
	(95 % CI)	(95 % CI)	(95 % CI)	(95 % CI)	(95 % CI)	(95 % CI)	(95 % CI)	(95 % CI)
Catheter colonization								
Skin + NCs	80.0	55.2	28.6	92.5	59.8	18.3	1.79	0.36
	(56.4–100)	(42.6–67.9)	(13.7–4434)-	(83.0–100)	(48.5–71.0)	(9.3–27.3)	(1.24–2.58)	(0.13–1.02)
Skin	66.7	82.0	45.4	91.7	79.3	18.3	3.72	0.41
	(39.5–93.9)	(72.2–92.0)	(22.4–68.5)	(83.8–99.5)	(69.9–88.6)	(9.3–27.3)	(1.99–6.96)	(0.20–0.84)
NCs	40.0	67.2	21.4	83.3	62.2	18.3	1.22	0.89
	(11.9–68.1)	(55.2–79.2)	(4.24–38.4)	(72.5–94.2)	(51.0–73.3)	(9.3–27.3)	(0.60–2.47)	(0.57–1.40)
C-RBSI								
Skin + NCs	100	50.6	7.1	100	52.4	3.7	2.03	NA
	(83.3–100)	(39.0–62.3)	(0.0–16.1)	(98.7–100)	(41.0–63.9)	(0.0–8.3)	(1.62–2.53)	
Skin	100	75.9	13.6	100	76.8	3.7	4.16	NA
	(83.3–100)	(65.9–86.0)	(0.0–30.2)	(99.2–100)	(67.0–86.6)	(0.0–8.3)	(2.81–6.15)	
NCs	66.7	67.0	7.1	98.1	67.0	3.7	2.03	0.50
	(0.0–100)	(56.0–78.0)	(0.0–18.5)	(93.6–100)	(56.3–77.8)	(0.0–8.3)	(0.86–4.79)	(0.10–2.48)

*S* sensitivity, *SP* specificity, *PPV* positive predictive value, *NPV* negative predictive value, *LR+* positive likelihood ratio, *LR−* negative likelihood ratio, *CI* confidence interval, *NA* not applicable, *NC* needleless connector, *C-RBSI* catheter-related bloodstream infection

The microorganisms isolated from the colonized CVCs are detailed in Table 3.

**Table 3:** Microorganisms isolated in colonized catheters

Catheter tip	Skin + NC	Skin	NC
*Staphylococcus epidermidis*	*Staphylococcus epidermidis*	*Staphylococcus epidermidis*	*-*
*Staphylococcus epidermidis*	*Staphylococcus epidermidis*	*-*	*Staphylococcus epidermidis*
*Staphylococcus epidermidis*	*-*	*-*	*-*
*Proteus mirabilis*	*Proteus mirabilis*	*Proteus mirabilis*	*-*
*Proteus mirabilis*	*Proteus mirabilis*	*Proteus mirabilis*	*-*
*Staphylococcus epidermidis*	*Staphylococcus epidermidis*	*Staphylococcus epidermidis*	*Staphylococcus epidermidis*
	*Staphylococcus haemolyticus*	*-*	*Staphylococcus haemolyticus*
	*Staphylococcus chromogenes*	-	*Staphylococcus chromogenes*
*Staphylococcus epidermidis*	*Staphylococcus epidermidis*	*Staphylococcus epidermidis*	-
*Staphylococcus epidermidis*	*Staphylococcus epidermidis*	*Staphylococcus epidermidis*	*Staphylococcus epidermidis*
*Staphylococcus epidermidis*	*Staphylococcus epidermidis*	*Staphylococcus epidermidis*	*-*
CoNS	*-*	*-*	*-*
*Staphylococcus epidermidis*	*Staphylococcus epidermidis*	*Staphylococcus epidermidis*	*Staphylococcus epidermidis*
	*Moraxella osloensis*	*-*	*Moraxella osloensis*
	*Klebsiella pneumoniae*	*Klebsiella pneumoniae*	*-*
*Candida albicans*	*Staphylococcus hominis*	*-*	*Staphylococcus hominis*
*Staphylococcus epidermidis*	*Staphylococcus epidermidis*	*Staphylococcus epidermidis*	*-*
	*Staphylococcus aureus*		*Staphylococcus aureus*
	*Staphylococcus saprophyticus*		*Staphylococcus saprophyticus*
*Staphylococcus epidermidis*	*Staphylococcus epidermidis*	*Staphylococcus epidermidis*	*-*
*Staphylococcus hominis*	*-*	*-*	*-*

*NC* needleless connector, *CoNS* coagulase-negative staphylococci

A Kaplan-Meier analysis showed that the earlier a superficial culture was positive, the greater the chance of catheter tip colonization (*P* = 0.19). Of the 15 colonized catheters, the concordance (identification of genus and species) between superficial cultures (skin or lumens or both) and colonized tips was 73.3 %.

## Discussion

Negative cultures from the skin surrounding the catheter entry site and from flushed catheter NCs are good predictors of the absence of catheter tip colonization. In patients with bacteremia, the negativity of skin and NC cultures practically rules out the causal role of the catheter in bloodstream infection.

C-RBSI is a major nosocomial infection with high rates of morbidity and mortality, especially in MHS-ICU patients [14, 15]. Colonization of the catheter tip is considered a pre-requisite for the development of C-RBSI, which occurs by migration of microorganisms to the catheter tip along the inner or the outer surface [3, 4]. In clinical practice, more than 50 % of the catheter tips withdrawn with suspected C-RBSI actually prove to be culture-negative in the microbiology department; that is, non-colonized catheters are withdrawn early and unnecessarily [16].

Several authors, including our group, have demonstrated that negative superficial cultures of the skin surrounding the catheter insertion site and catheter hubs ruled out catheter tip colonization in MHS-ICU, oncology, and hemodialysis patients, thus avoiding unnecessary withdrawals of the catheter [3, 6, 7]. Catheter hub cultures are obtained by rubbing swabs on the inside of the hubs and therefore carry a potential risk of dislodging microorganisms [9–11].

Considering that NCs are replaced regularly to decrease the possibility of colonization, we thought that they could be used as an alternative diagnostic method to hub cultures and thus would enable us to avoid unnecessary manipulation. We recently demonstrated that NCs were capable of ruling out catheter tip colonization by culturing their outer surface after sonication [17]. However, we did not assess the yield of the inner surface of NCs combined with skin culture for prediction of catheter colonization and C-RBSI. In the present study, we showed that negative NCs and superficial cultures had a high negative predictive value for catheter colonization and practically ruled out the catheter as the source of bacteremia.

The main limitations of our study were its small sample size, the need to obtain a high number of NC cultures (with the consequent high workload and costs), and the fact that our results cannot be immediately extrapolated to populations other than MHS-ICU patients. Our findings may be interpreted with caution because of the extrapolation of the microbiological results to the clinical setting. Whether clinicians are supposed to use this information to decide that it is appropriate to maintain a central line that could otherwise be removed or to replace a colonized CVC with a new one is a point that should be assessed in future clinical studies.

Clinical trials are required to verify whether early withdrawal of catheters in patients with positive superficial cultures could contribute to the objective of “zero tolerance” of C-RBSI in ICUs, especially in patients with problems of vascular accessibility, coagulopathy, or severe respiratory disease to avoid the CVC removal and the risk of mechanical complications during the new canalization. In addition, the value of these cultures needs to be assessed in terms of the number of sets of NCs and catheters obtained in each patient.

## Conclusions

If our data are confirmed by other groups, surveillance of catheter colonization (using NCs and skin cultures) could help to identify patients at risk of C-RBSI. These methods might help identify patients at low risk for C-RBSI.

## Key message

- Closed NCs may serve as a safer alternative diagnostic procedure to predict catheter colonization in MHS-ICU patients.

## Abbreviations

CFU: Colony-forming unit
CI: Confidence interval
CVC: Central venous catheter
C-RBSI: Catheter-related bloodstream infection
ICU: Intensive care unit
MHS-ICU: Major heart surgery intensive care unit
NC: Needleless connector
SD: Standard deviation
